# Cooperation of σ–π and σ*–π* Conjugation in the UV/Vis and Fluorescence Spectra of 9,10-Disilylanthracene

**DOI:** 10.3390/molecules27072241

**Published:** 2022-03-30

**Authors:** Soichiro Kyushin, Yuya Suzuki

**Affiliations:** Division of Molecular Science, Graduate School of Science and Technology, Gunma University, Kiryu 376-8515, Gunma, Japan; osicl-0903@yahoo.co.jp

**Keywords:** anthracene, fluorescence spectrum, σ–π conjugation, σ*–π* conjugation, silyl group, UV/Vis spectrum

## Abstract

In 1996, we reported that silyl groups of 9,10-disilylanthracenes significantly affect the UV/Vis and fluorescence spectra. Although the results indicate that the silyl groups have strong electronic effects on anthracene, the details of the mechanisms responsible for this have not yet been clarified. This article describes the analysis of the UV/Vis and fluorescence spectra of 9,10-bis(diisopropylsilyl)anthracene by theoretical calculations. This study reveals that π conjugation of anthracene is extended by cooperation of σ–π and σ*–π* conjugation between the silyl groups and anthracene. This effect increases the transition moment of the π–π* transition of anthracene. As a result, the molecular extinction coefficient of the ^1^L_a_ band and the fluorescence quantum yield are increased.

## 1. Introduction

Studies on the electronic effects of silyl groups go back to the middle of the last century. The electronic effects of silyl groups were recognized by unusual accelerations of the reactions of some organosilicon compounds [1]. Later, the electronic effects were observed by UV [2] and photoelectron [3] spectroscopies. Today, σ–π conjugation between σ(Si–C or Si–Si) and π orbitals [4], σ*–π* conjugation between σ*(Si–C) and π* orbitals [5] are well known. These electronic effects are considered to be caused by relatively high energy levels of the σ orbitals, relatively low energy levels of the σ* orbitals and large lobes of the σ and σ* orbitals.

**Figure 1:**
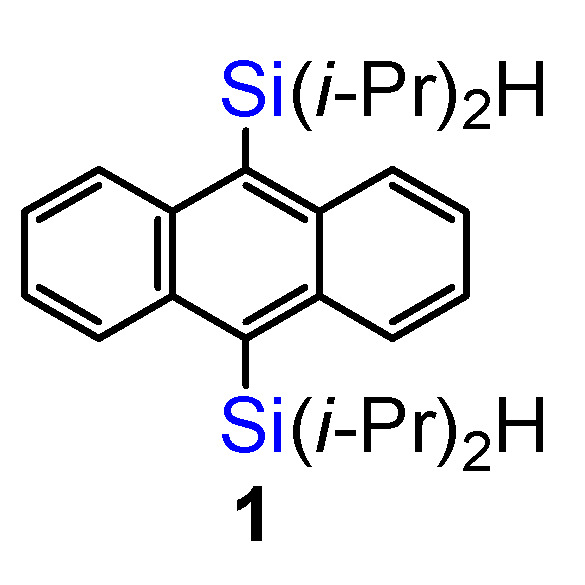
Structure of **1**.

In 1996, we reported that silyl groups of 9,10-bis(diisopropylsilyl)anthracene (**1**, Figure 1) and related 9,10-disilylanthracenes significantly affect the UV/Vis and fluorescence spectra [6]. The absorption maximum of the ^1^L_a_ band in the UV/Vis spectrum of **1** (*λ*_max_ = 399 nm (*ε* = 14200 mol^–1^ L cm^–1^)) shows a considerable bathochromic shift with the large molecular extinction coefficient compared with that of anthracene (*λ*_max_ = 374 nm (*ε* = 8000 mol^–1^ L cm^–1^)). The fluorescence quantum yield of **1** (*Φ*_f_ = 0.90 (room temperature)) is much larger than that of anthracene (*Φ*_f_ = 0.32 (room temperature)). These results indicate that the silyl groups affect both the HOMO and LUMO. Similar results have also been reported by other groups [7,8,9,10]. After this finding, UV/Vis, fluorescence and other measurements of various silyl-substituted aromatic compounds have been reported, showing electronic effects of silyl groups [11,12,13,14,15,16,17,18,19,20,21,22,23,24,25,26,27,28,29].

In spite of numerous studies on the UV/Vis and fluorescence spectra of silyl-substituted aromatic compounds, the effects of silyl groups have not yet been fully determined. On the other hand, studies on the UV/Vis spectra of unsubstituted aromatic compounds have been fruitful in recent years [30]. Studies on the substituent effects of silyl groups are important not only for the development of fundamental organosilicon chemistry, but also from the viewpoint of optoelectronic applications to dyes and luminescent materials. We report herein a theoretical analysis of the electronic effects of the silyl groups of **1** at the TD-DFT B3LYP/6-31G(d) level. We found that cooperation of electronic effects of the silyl groups in the HOMO and LUMO is important in the UV/Vis and fluorescence spectra of **1**.

## 2. Results and Discussion

### 2.1. Measurement of the UV/Vis and Fluorescence Spectra of ***1*** and Anthracene

**Figure 2:**
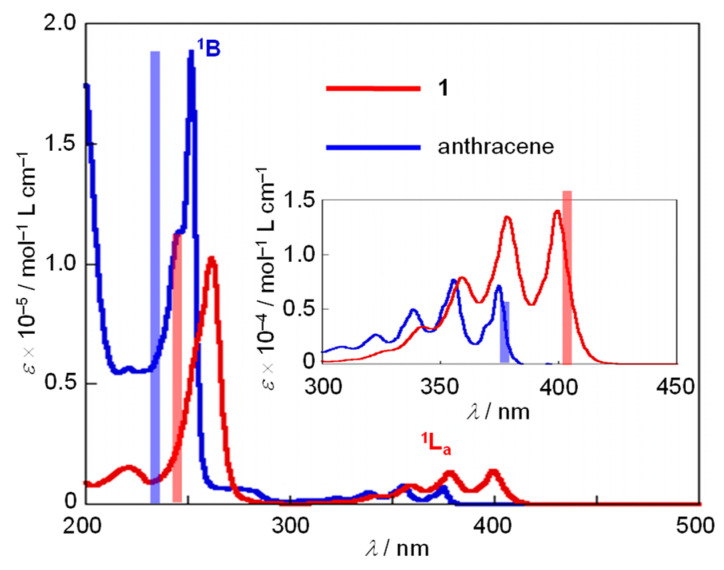
UV/Vis spectra of **1** and anthracene in hexane at room temperature. The pale red (**1**) and blue (anthracene) bars denote absorption calculated by using the optimized structures of the S_0_ states of **1** and anthracene at the TD-DFT B3LYP/6-31G(d) level.

In our previous paper [6], we compared the ^1^L_a_ band of **1** at 300–450 nm with that of anthracene [31,32,33,34]. To get a big picture, the whole UV/Vis spectra of **1** and anthracene were measured (Figure 2). The intense absorption band at ca. 250 nm and the vibration bands at 300–380 nm of anthracene had already been assigned to the ^1^B and ^1^L_a_ bands, respectively [35,36,37]. The UV/Vis spectrum of **1** has three features: (1) The molecular extinction coefficient of the ^1^B band (*λ*_max_ = 262 nm (*ε* = 102,000 mol^–1^ L cm^–1^)) is much smaller than that of anthracene (*λ*_max_ = 252 nm (*ε* = 190,000 mol^–1^ L cm^–1^)); (2) On the other hand, the molecular extinction coefficient of the ^1^L_a_ band (*λ*_max_ = 400 nm (*ε* = 14,000 mol^–1^ L cm^–1^)) is larger than that of anthracene (*λ*_max_ = 375 nm (*ε* = 7200 mol^–1^ L cm^–1^)); and (3) The ^1^B and ^1^L_a_ bands of **1** show bathochromic shifts compared with those of anthracene.

**Figure 3:**
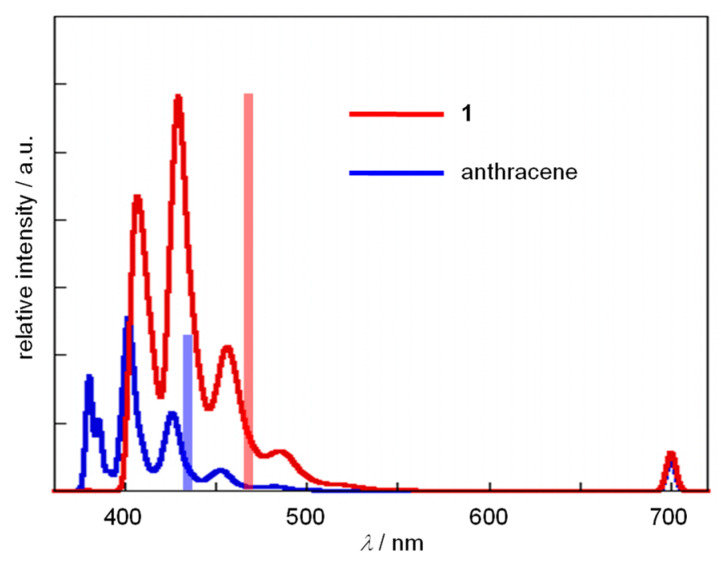
Fluorescence spectra of **1** and anthracene in 3-methylpentane at 77 K. The pale red (**1**) and blue (anthracene) bars denote fluorescence calculated by using the optimized structures of the S_1_ states of **1** and anthracene at the TD-DFT B3LYP/6-31G(d) level.

We also measured the fluorescence spectra of **1** and anthracene at 77 K (Figure 3). The fluorescence of anthracene is observed at 370–520 nm, whereas the fluorescence bands of **1** shift bathochromically to 400–570 nm. These fluorescence spectra resemble those measured at room temperature [6], although the vibrational fine structures are sharp, and peaks are more separated. The fluorescence quantum yield of **1** (*Φ*_f_ = 0.98 (77 K)) is much larger than that of anthracene (*Φ*_f_ = 0.34 (77 K)) [38]. The phosphorescence of **1** and anthracene could not be observed. The phosphorescence quantum yield of anthracene has been reported to be very small (*Φ*_p_ = 0.0003 [38]). The data of the UV/Vis and fluorescence spectra of **1** and anthracene are summarized in Table 1.

### 2.2. Theoretical Analysis of the UV/Vis Spectra of ***1*** and Anthracene

**Figure 4:**
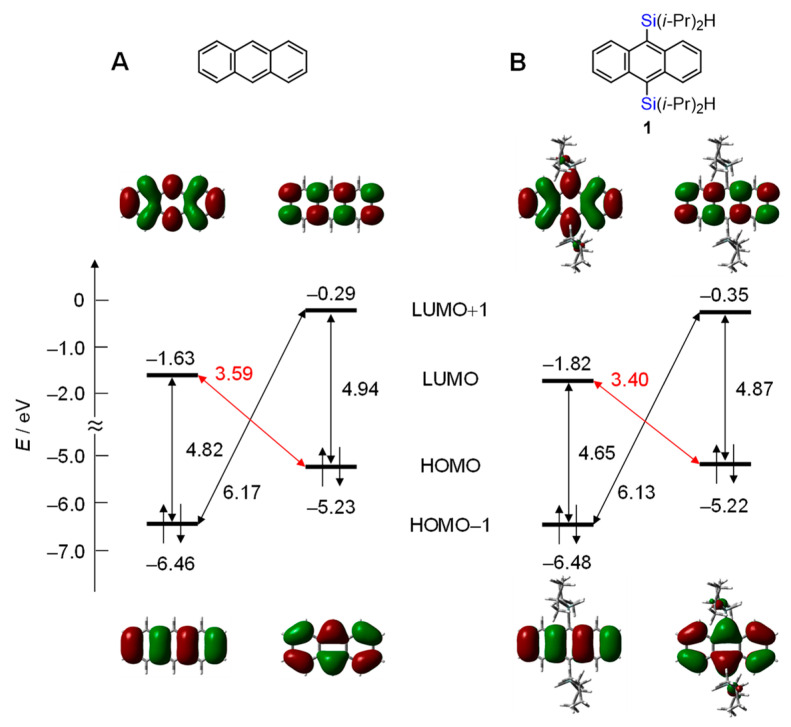
The HOMO, HOMO–1, LUMO, LUMO+1 and their energy levels of **1** (**B**) and anthracene (**A**) calculated at the B3LYP/6-31G(d) level.

The HOMO, HOMO–1, LUMO and LUMO+1 of **1** and anthracene are shown in Figure 4. The energy levels of the HOMO and HOMO–1 of **1** are similar to those of anthracene. However, the HOMO of **1** clearly shows out-of-phase interaction between σ(Si–C) orbitals and a π orbital of the anthracene moiety (σ–π conjugation, Figure S1) [11,15,16,25,28]. The effect of the σ–π conjugation on the energy levels is small probably because the distance between the σ(Si–C) and π orbitals is relatively large. The energy levels of the LUMO and LUMO+1 of **1** are lower than those of anthracene. Notably, the LUMO clearly shows in-phase interaction between the σ*(Si–C) and π* orbitals of anthracene (σ*–π* conjugation, Figure S1) [5,15,16,25,28]. The relatively small distance between the σ*(Si–C) and π* orbitals makes the energy level of the LUMO low. TD-DFT calculations show that the ^1^L_a_ bands of **1** and anthracene are mainly due to the HOMO-to-LUMO transitions with the configuration-interaction (CI) coefficient of 0.70 (Table S3). The energy gaps between the HOMO and LUMO are 3.40 (**1**) and 3.59 eV (anthracene). The smaller energy gap of **1** is in accordance with the bathochromic shift of the ^1^L_a_ band compared with that of anthracene.

The transition moments of the HOMO-to-LUMO transitions of **1** and anthracene are 1.46 and 0.85 a.u., respectively. The large transition moment of **1** corresponds to the large molecular extinction coefficient of the ^1^L_a_ band of **1** (Figure 2) [6]. As the HOMO-to-LUMO transition mainly contributes to the ^1^L_a_ band, the transition moment can be expressed by

**Scheme 1:**
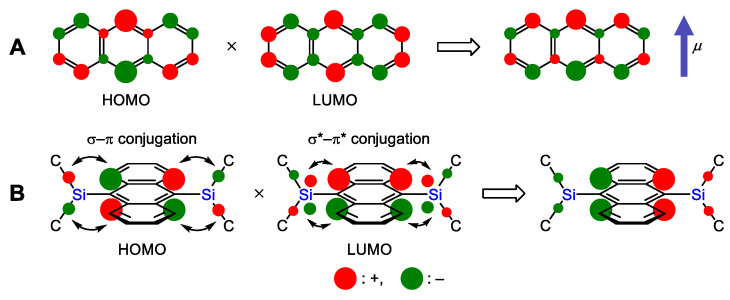
Multiplication of the HOMO and LUMO of anthracene and the transition moment of the HOMO–LUMO transition (**A**). The effect of the silyl groups on the transition moment is shown in the (**B**).

*μ* ≈ ∫*ψ*_HOMO_***M****ψ*_LUMO_ d*τ* (1)

where *ψ*_HOMO_ and *ψ*_LUMO_ are the wave functions of the HOMO and LUMO, respectively. ***M*** is the operator of the dipole moment [39,40,41,42]. The multiplication of *ψ*_HOMO_ and *ψ*_LUMO_ is shown in Scheme 1. The σ–π and σ*–π* conjugation give positive (or negative) areas just outside of the positive (or negative) areas of the π orbital of the anthracene ring. This extension of areas with the same signal occurs at the 9,10-positions of anthracene. As the transition moment of anthracene is in the direction of the short molecular axis, this extension increases the transition moment. Therefore, cooperation of the σ–π and σ*–π* conjugation makes the molecular extinction coefficient of the ^1^L_a_ band of **1** larger than that of anthracene.

**Figure 5:**
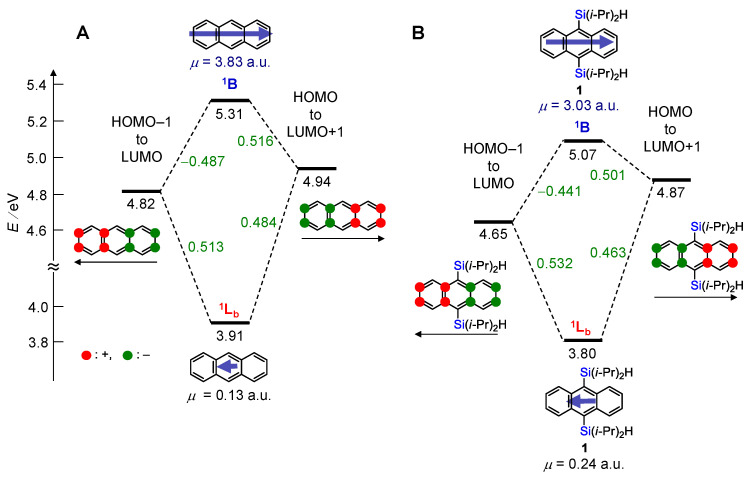
Configuration interaction between transitions for the ^1^B and ^1^L_b_ bands of **1** (**B**) and anthracene (**A**) calculated at the TD-DFT B3LYP/6-31G(d) level. Energy levels were calculated at the B3LYP/6-31G(d) level. The structures with positive and negative circles show the products of orbital pairs representing the transition. The arrows under the structures denote transition moments.

The TD-DFT calculation showed that the ^1^B band of anthracene is formed by the CI between the HOMO–1-to-LUMO and HOMO-to-LUMO+1 transitions; other transitions do not contribute to this band (Figure 5A). Both transitions have transition moments in the direction of the long molecular axis of anthracene. These two transition moments are enhanced by each other, as shown by the CI coefficients, giving the large total transition moment of 3.83 a.u. Similarly, the ^1^B band of **1** is formed by the CI between the HOMO–1-to-LUMO and HOMO-to-LUMO+1 transitions (Figure 5B). Although the transition moments of these transitions are enhanced by each other, the total transition moment (3.03 a.u.) is significantly smaller than that of anthracene. The squares of the transition moments as well as the oscillator strengths of the ^1^B bands of **1** (*μ*^2^ = 9.2 a.u.^2^, *f* = 1.14) and anthracene (*μ*^2^ = 14.7 a.u.^2^, *f* = 1.91) are nearly in line with the molecular extinction coefficients of the ^1^B bands (**1**: *ε* = 102000 mol^−1^ L cm^−1^, anthracene: *ε* = 190000 mol^−1^ L cm^−1^). These results indicate that the difference of the intensity of the ^1^B bands could be ascribed mainly to the electron transition, although the effect of vibronic coupling could not be completely excluded.

**Figure 6:**
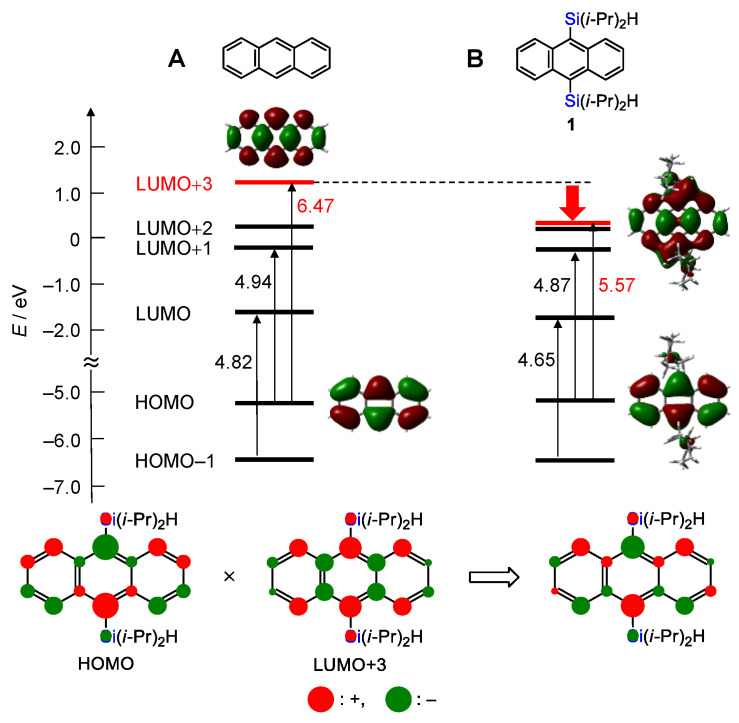
Comparison of the HOMO-to-LUMO+3 transitions of **1** (**B**) and anthracene (**A**) calculated at the B3LYP/6-31G(d) level. The product of the HOMO and LUMO+3 of **1** is shown at the **bottom**.

The relatively small transition moment of the ^1^B band of **1** compared with that of anthracene is ascribed to contribution of the HOMO-to-LUMO+3 transition (Figure 6A,B and Table S3). The CI coefficients of the ^1^B band of anthracene are −0.487 (HOMO–1-to-LUMO) and 0.516 (HOMO-to-LUMO+1), whereas those of **1** are smaller, i.e.,: –0.441 (HOMO–1-to-LUMO) and 0.501 (HOMO-to-LUMO+1). Instead, the HOMO-to-LUMO+3 transition contributes with the CI coefficient of 0.186. The HOMO-to-LUMO+3 transition of anthracene has a high energy (6.47 eV) and does not interact with the HOMO–1-to-LUMO (4.82 eV) and HOMO-to-LUMO+1 (4.94 eV) transitions. However, the energy level of the LUMO+3 of **1** is very low because of effective σ*–π* conjugation between σ*(Si–C) orbitals and a π* orbital of anthracene (Figure 6). As a result, the HOMO-to-LUMO+3 transition of **1** has a small energy (5.57 eV) and interacts with the HOMO–1-to-LUMO (4.65 eV) and HOMO-to-LUMO+1 (4.87 eV) transitions. The HOMO-to-LUMO+3 transition does not have a transition moment in the direction of the long molecular axis (Figure 6 (bottom)). The relatively reduced contribution of the HOMO–1-to-LUMO and HOMO-to-LUMO+1 transitions to the ^1^B band leads to the relatively small transition moment.

The discussion of the ^1^L_a_ and ^1^B bands shows that the silyl groups affect these bands in different manners. In the ^1^L_a_ band, the silyl groups “directly” enhance the transition moment of anthracene. On the other hand, the silyl groups allow another transition to contribute to the ^1^B band. This effect reduces contribution of the original transitions of anthracene and weakens the transition moment. This is an “indirect” effect of the silyl groups.

The TD-DFT calculations also show that the ^1^L_b_ bands of **1** and anthracene must be present at 326 and 317 nm, respectively. However, as the transition moments (**1**: 0.24 a.u., anthracene: 0.13 a.u.) are much smaller than those of the ^1^L_a_ bands (**1**: 1.46 a.u., anthracene: 0.85 a.u.), the ^1^L_b_ bands are hidden by the ^1^L_a_ bands in both compounds.

### 2.3. Optimized Structures of the Excited Singlet States of ***1*** and Anthracene

**Figure 7:**
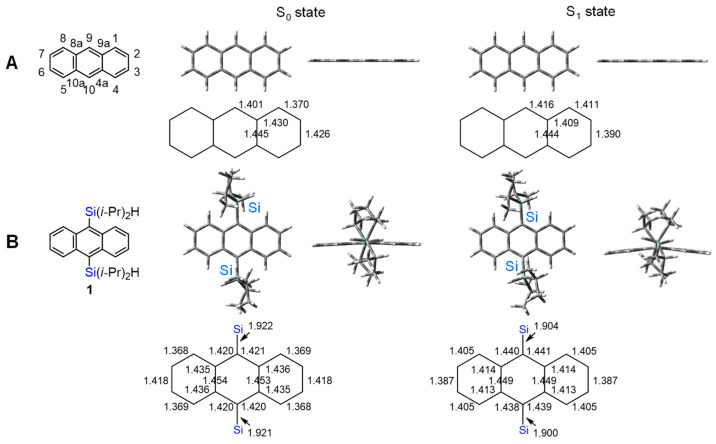
Top and side views and bond lengths (Å) of the optimized structures of the S_0_ and S_1_ states of **1** (**B**) and anthracene (**A**). The structures of the S_0_ and S_1_ states were calculated at the B3LYP/6-31G(d) and TD-DFT B3LYP/6-31G(d) levels, respectively.

The optimized structures of the excited singlet states (S_1_) of **1** and anthracene were calculated at the TD-DFT B3LYP/6-31G(d) level and compared with those of the ground states (S_0_). The structures and structural parameters are shown in Figure 7. The S_0_ and S_1_ states of anthracene have completely planar structures with the *D*_2*h*_ symmetry. The anthracene moiety of the S_0_ state of **1** has a slightly bent structure with a fold angle (4.7°) between the C9–C9a–C1–C2–C3–C4–C4a–C10 and C10–C10a–C5–C6–C7–C8–C8a–C9 planes. The fold angle increases in the case of the S_1_ state (12.6°). The bent structure of the S_1_ state of **1** could be explained by the effects of molecular orbitals and steric hindrance. By excitation from the S_0_ state to the S_1_ state, an electron in the HOMO is transferred to the LUMO. The p orbitals at the 9,10-positions, which have the largest coefficients in the HOMO, interact with the adjacent p orbitals with the in-phase mode (Figure 4). In contrast, the p orbitals at the 9,10-positions in the LUMO interact with the adjacent p orbitals in the out-of-phase mode. The π interaction between the 9,10- and adjacent carbon atoms is weakened by the excitation. In addition, the steric hindrance between the bulky diisopropylsilyl groups and the four hydrogen atoms at the *peri*-positions is present in the planar structure. A combination of these electronic and steric effects results in the bent structure of the anthracene moiety.

The optimized structure of the S_1_ state of **1** has another feature. The C(1)–C(2), C(3)–C(4), C(5)–C(6) and C(7)–C(8) bonds (1.405 Å) and the C(9)–C(9a), C(4a)–C(10), C(10)–C(10a) and C(8a)–C(9) bonds (1.438–1.441 Å) are longer than the corresponding bonds (1.368–1.369 Å and 1.420–1.421 Å, respectively) of the S_0_ state, whereas the C(2)–C(3) and C(6)–C(7) bonds (1.387 Å) and the C(1)–C(9a), C(4)–C(4a), C(5)–C(10a) and C(8)–C(8a) bonds (1.413–1.414 Å) of the S_1_ state are shorter than the corresponding bonds (1.418 Å and 1.435–1.436 Å, respectively) of the S_0_ state. These structural changes are due to the electron transition from the bonding (or antibonding) π orbital of the HOMO to the antibonding (or bonding) π* orbital of the LUMO. Furthermore, the Si–C(9) (1.904 Å) and Si–C(10) (1.900 Å) bonds of the S_1_ state of **1** are shortened compared with the corresponding bonds (1.922 and 1.921 Å, respectively) of the S_0_ state. The shortening could be explained by σ*–π* conjugation between the silicon and C(9 or 10) atoms in the LUMO.

### 2.4. Theoretical Analysis of the Fluorescence Spectra of ***1*** and Anthracene

**Figure 8:**
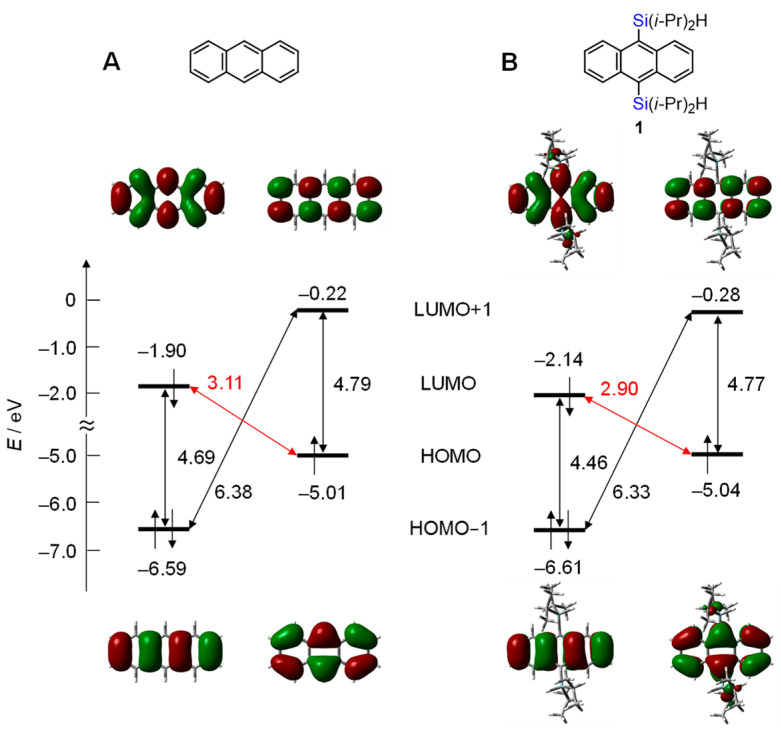
The HOMO, HOMO−1, LUMO, LUMO+1 and their energy levels of the S_1_ states of **1** (**B**) and anthracene (**A**) calculated at the B3LYP/6-31G(d) level.

The S_1_ states of **1** and anthracene emit fluorescence and are deactivated to the S_0_ states according to the Franck–Condon principle [43,44]. The frontier orbitals and energy levels of the S_1_ states of **1** and anthracene calculated by using the optimized S_1_ structures are shown in Figure 8. Although the energy level of the HOMO of **1** is similar to that of anthracene, lobes of the HOMO show σ–π conjugation (Figure S2). The energy level of the LUMO of **1** is lower than that of anthracene due to σ*–π* conjugation. The energy gap between the HOMO and the LUMO of the S_1_ state of **1** (2.90 eV) is smaller than that of anthracene (3.11 eV). This result is in accord with the bathochromic shift of the fluorescence bands of **1** compared with those of anthracene (Figure 3).

The transition moment of the electron transition from the LUMO to the HOMO of the S_1_ state was calculated. The transition moment of **1** (1.55 a.u.) is larger than that of anthracene (0.93 a.u.) by cooperation of σ–π and σ*–π* conjugation, similar to the UV/Vis spectra (Scheme 1, Figure S2). The squares of the transition moments of **1** (*μ*^2^ = 2.42 a.u.^2^) and anthracene (*μ*^2^ = 0.86 a.u.^2^) are nearly proportional to the fluorescence quantum yield of **1** (*Φ*_f_ = 0.90) and anthracene (*Φ*_f_ = 0.32) [6]. It is noted that the transition moment of **1** is larger than that of the electron transition from the HOMO to the LUMO of the S_0_ state of **1** (1.46 a.u.). The larger transition moment of the S_1_ state is ascribed to the shortening of the Si–C bonds. The short Si–C bonds lead to effective σ–π and σ*–π* conjugation, as shown by larger lobes of the σ(Si–C) and σ*(Si–C) orbitals than those of the S_0_ state (Figures S1 and S2). As the lobes of the σ(Si–C) and σ*(Si–C) orbitals become larger, the transition moment becomes larger, as shown in Scheme 1.

## 3. Materials and Methods

Compound **1** was prepared according to the reported procedure [6]. Anthracene (Merck, Darmstadt, Germany, 99.5%) was purchased and used without further purification.

UV/Vis spectra were recorded on a V-570 UV/VIS/NIR spectrophotometer (JASCO, Hachioji, Japan). Fluorescence spectra were obtained on an F-4500 fluorescence spectrophotometer (Hitachi, Tokyo, Japan). Fluorescence quantum yields were determined by using a C9920-02 absolute PL quantum yield measurement system (Hamamatsu Photonics, Hamamatsu, Japan).

All theoretical calculations were performed by using Gaussian 09 [45] and 16 [46] on a PRIMERGY RX300 system (Fujitsu, Tokyo, Japan) of the Research Center for Computational Science, Japan. The structures of the S_0_ and S_1_ states of **1** and anthracene were optimized at the B3LYP/6-31G(d) and TD-DFT B3LYP/6-31G(d) levels, respectively, and the optimization was confirmed by frequency calculations. The results are summarized in Tables S1 and S2. Transition properties of **1** and anthracene were calculated at the TD-DFT B3LYP/6-31G(d) level by using the optimized structures. The results are summarized in Tables S3 and S4.

## 4. Conclusions

This study reveals substituent effects of the silyl groups on the UV/Vis and fluorescence spectra of **1**. Cooperation of the σ–π conjugation in the HOMO and the σ*–π* conjugation in the LUMO leads to the bathochromic shifts of the UV/Vis and fluorescence bands. Furthermore, the decrease of the ^1^B band, the increase of the ^1^L_a_ band, and the increase of the fluorescence quantum yield are rationalized. This is the first step toward clarifying the substituent effects of silyl groups on the optical properties of silyl-substituted aromatic compounds. To develop this work, further studies on the effects of silyl groups at various substitution positions of aromatic compounds and the effects of substituents on a silyl group are necessary. These studies will be undertaken in the near future.

## Figures and Tables

**Table 1:** Photophysical parameters of **1** and anthracene.

Compound	*λ*_a_/nm ^1^ (*ε*/mol^–1^ L cm^–1^)	*λ*_f_/nm ^2^
**1**	221 (15,400) 262 (102,000) 343 (3400) 359 (7900) 378 (13,500) 400 (14,000)	406 429 456 485
anthracene	252 (190,000) 323 (2700) 339 (5000) 356 (7700) 375 (7200)	380 402 426 453 482

^1^ Measured in hexane at room temperature. ^2^ Measured in 3-methylpentane at 77 K.

## Data Availability

The data presented in this study are contained within the article and are also available in the Supplementary Materials.

## Supplementary Materials

The following are available online at https://www.mdpi.com/article/10.3390/molecules27072241/s1, Tables S1 and S2: Atomic coordinates of the optimized structures of the S_0_ and S_1_ states of **1** and anthracene, Tables S3 and S4: Transition energies, wavelengths and oscillator strengths of the transitions of the S_0_ and S_1_ states of **1** and anthracene, Figures S1 and S2: Side views of the frontier orbitals of the S_0_ and S_1_ states of **1**, Figure S3: Calculated vibrationally resolved UV/Vis and fluorescence spectra of anthracene.

## Sample Availability

Samples of the compounds are not available from the authors.
