# Supplementary material for: Cooperation of σ–π and σ*–π* Conjugation in the UV/Vis and Fluorescence Spectra of 9,10-Disilylanthracene
Source: Molecules. 2022 Mar 30;27(7):2241. doi: 10.3390/molecules27072241 (PMC9000373; doi:10.3390/molecules27072241)
Supplement: Supplementary file 1 [file molecules-27-02241-s001.zip › molecules-1534198-supplementary.pdf]

Supplementary Materials

Cooperation of σ–π and σ*–π* Conjugation in the UV/Vis and Fluorescence Spectra of 9,10-Disilylanthracene

Soichiro Kyushin * and Yuya Suzuki

Contents

1. Theoretical calculations

1. Theoretical calculations

All theoretical calculations were performed by using Gaussian 09 [45] and 16 [46] on a Fujitsu PRIMERGY RX300 system of the Research Center for Computational Science, Japan. The structures of the S_0 and S_1 states of **1** and anthracene were optimized at the B3LYP/6-31G(d) and TD-DFT B3LYP/6-31G(d) levels, respectively, and the optimization was confirmed by frequency calculations. The results are summarized in Tables S1 and S2. Transition properties of **1** and anthracene were calculated at the TD-DFT B3LYP/6-31G(d) level by using the optimized structures. The results are summarized in Tables S3 and S4. Vibrationally resolved UV/Vis and fluorescence spectra of anthracene were calculated by using the frequency data of the optimized structures of the S_0 and S_1 states. The results are shown in Figure S3. Unfortunately, similar calculations of vibrationally resolved UV/Vis and fluorescence spectra of **1** failed.

Table S1. Atomic coordinates of the optimized structures of the S_0 states of **1** and anthracene.

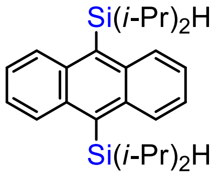

1

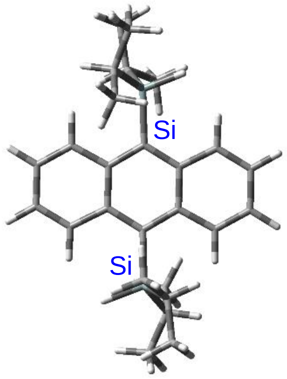

Atomic Type	Coordinates (Angstroms) X	Y	Z
C	-0.1053590	-3.7022510	-0.5498870
C	-0.9712340	-2.6511180	-0.4133160
C	-0.5258440	-1.3072810	-0.1718110
C	0.9078910	-1.0836710	-0.0886570
C	1.7687500	-2.2234690	-0.2306360
C	1.2925970	-3.4872880	-0.4503490
C	-1.4337660	-0.2262350	-0.0080050
C	1.4433290	0.2114400	0.1402690
C	0.5346970	1.2771940	0.3775410
C	-0.8994170	1.0539200	0.2973740
C	-1.7597510	2.1777330	0.5366590
H	-2.8324540	2.0404020	0.4738470
C	-1.2830760	3.4212400	0.8508490

C	0.1156190	3.6331580	0.9484880
C	0.9816110	2.5994390	0.7152520
H	-0.4901840	-4.7031250	-0.7259230
H	-2.0329930	-2.8417700	-0.4814810
H	2.8395750	-2.0866640	-0.1370850
H	1.9816590	-4.3225640	-0.5412670
H	-1.9733540	4.2412690	1.0292960
H	0.4997430	4.6156140	1.2093120
H	2.0439790	2.7823140	0.7977500
Si	3.3437660	0.4620070	0.0112040
H	3.6783610	1.9034570	0.1796020
Si	-3.3393200	-0.4283340	-0.1564660
H	-3.6671080	-1.8042100	-0.6226590
C	3.9182170	0.0338480	-1.7694040
H	3.6143420	-0.9996070	-1.9847260
C	5.4477280	0.1193190	-1.9280720
H	5.9736630	-0.5880460	-1.2764910
H	5.7434320	-0.1059290	-2.9619640
H	5.8210590	1.1255540	-1.6979020
C	3.2142310	0.9455360	-2.7936880
H	2.1239250	0.8515680	-2.7448480
H	3.4650980	2.0011450	-2.6282730
H	3.5245670	0.6930200	-3.8168120
C	4.3269250	-0.4527530	1.3917440
H	4.6282550	-1.4341330	0.9939320
C	3.4882180	-0.6979880	2.6607520
H	3.1368080	0.2459520	3.0965590
H	2.6068470	-1.3148730	2.4596900
H	4.0872300	-1.2065450	3.4284320
C	5.6160390	0.3175100	1.7446320
H	6.2034600	-0.2317190	2.4930030
H	6.2603230	0.4801940	0.8738570
H	5.3832310	1.3018760	2.1688120
C	-4.1360020	-0.2688020	1.5833900
H	-3.8060770	0.6876310	2.0132740
C	-4.1092430	0.7173800	-1.4990270
H	-4.3868180	1.6709920	-1.0256390
C	-3.6218970	-1.3904230	2.5079840
H	-3.9165410	-2.3801150	2.1361120
H	-2.5300950	-1.3809720	2.5987060
H	-4.0401590	-1.2841200	3.5181190
C	-5.6752710	-0.2606280	1.5440960
H	-6.0889800	-0.1966640	2.5599120

H	-6.0706650	0.5891260	0.9761400
H	-6.0725020	-1.1780220	1.0910800
C	-5.3992670	0.0905440	-2.0684360
H	-6.1473590	-0.1131290	-1.2950140
H	-5.8608320	0.7614060	-2.8057310
H	-5.1858190	-0.8577750	-2.5765540
C	-3.1259760	1.0281460	-2.6441540
H	-2.2371130	1.5605710	-2.2927950
H	-2.7876560	0.1093710	-3.1403410
H	-3.6095810	1.6501040	-3.4097680

Framework group C_1, energy: -1592.68649600 a.u.

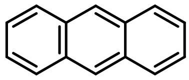

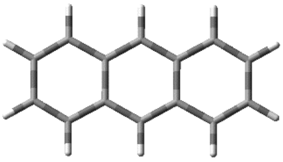

Atomic Type	Coordinates (Angstroms) X	Y	Z
C	0.0000000	3.6606800	0.7131450
C	0.0000000	2.4795300	1.4070360
C	0.0000000	1.2239230	0.7226250
C	0.0000000	1.2239230	-0.7226250
C	0.0000000	2.4795300	-1.4070360
C	0.0000000	3.6606800	-0.7131450
C	0.0000000	0.0000000	1.4033770
C	0.0000000	0.0000000	-1.4033770
C	0.0000000	-1.2239230	-0.7226250
C	0.0000000	-1.2239230	0.7226250
C	0.0000000	-2.4795300	1.4070360
H	0.0000000	-2.4768690	2.4945800
C	0.0000000	-3.6606800	0.7131450
C	0.0000000	-3.6606800	-0.7131450
C	0.0000000	-2.4795300	-1.4070360
H	0.0000000	0.0000000	2.4917480
H	0.0000000	4.6073590	1.2466580
H	0.0000000	2.4768690	2.4945800
H	0.0000000	2.4768690	-2.4945800
H	0.0000000	4.6073590	-1.2466580
H	0.0000000	0.0000000	-2.4917480
H	0.0000000	-4.6073590	1.2466580

H	0.0000000	-4.6073590	-1.2466580
H	0.0000000	-2.4768690	-2.4945800

Framework group D_{2h}, energy: -539.53052354 a.u.

Table S2. Atomic coordinates of the optimized structures of the S_1 states of **1** and anthracene.

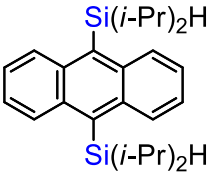

1

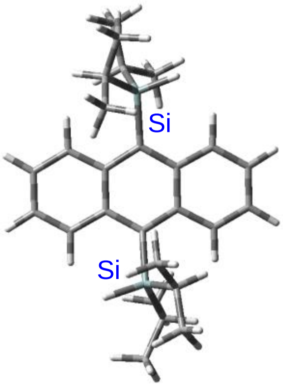

Atomic	Coordinates (Angstroms)		
Type	X	Y	Z
C	-0.1131910	3.7942920	-0.0975450
C	-0.9802800	2.6891990	-0.0578770
C	-0.5248930	1.3515600	-0.1168840
C	0.9054040	1.1375670	-0.2033120
C	1.7446740	2.2730220	-0.2541410
C	1.2544730	3.5888740	-0.2002890
C	-1.4235600	0.2253660	-0.1083590
C	1.4482860	-0.1941900	-0.2381630
C	0.5469720	-1.2858180	-0.4919810
C	-0.8839110	-1.0760460	-0.4080060
C	-1.7218740	-2.1929740	-0.6287110
H	-2.7963180	-2.0727230	-0.5508850
C	-1.2301530	-3.4686730	-0.9529250
C	0.1391790	-3.6586250	-1.0646370
C	1.0066560	-2.5774290	-0.8345250
H	-0.5244320	4.7984810	-0.0498820
H	-2.0423290	2.8797550	0.0240200
H	2.8151130	2.1327150	-0.3612420
H	1.9461300	4.4251800	-0.2413830
H	-1.9244910	-4.2880340	-1.1143530
H	0.5492090	-4.6309070	-1.3219450
H	2.0723410	-2.7471600	-0.9265310
Si	3.2837220	-0.4620140	0.1716060
H	3.5252760	-1.9231360	0.3486940
Si	-3.2856590	0.4056750	0.2437870
H	-3.5514610	1.7704020	0.7832630
C	3.6772510	0.3365400	1.8742840

H	3.4014630	1.3984080	1.8086880
C	5.1722500	0.2571780	2.2337900
H	5.8025900	0.7892020	1.5119550
H	5.3574970	0.7031370	3.2208910
H	5.5220230	-0.7823350	2.2798270
C	2.8159510	-0.3016740	2.9807480
H	1.7448940	-0.2002500	2.7744500
H	3.0335270	-1.3721600	3.0877980
H	3.0162400	0.1709880	3.9521830
C	4.4864970	0.1243340	-1.2198850
H	4.7931700	1.1526810	-0.9709800
C	3.8236090	0.1532970	-2.6099600
H	3.4766330	-0.8441880	-2.9085310
H	2.9577880	0.8226160	-2.6407850
H	4.5376270	0.4902560	-3.3740930
C	5.7595300	-0.7454360	-1.2507270
H	6.4652710	-0.3753810	-2.0072840
H	6.2835830	-0.7582970	-0.2890300
H	5.5204500	-1.7848740	-1.5074270
C	-4.2815860	0.2651090	-1.3982680
H	-3.9644360	-0.6707620	-1.8812940
C	-3.9044530	-0.7854480	1.6281070
H	-4.2142060	-1.7307890	1.1579220
C	-3.9083970	1.4209190	-2.3471150
H	-4.2055860	2.3912220	-1.9284190
H	-2.8315110	1.4598200	-2.5450370
H	-4.4211570	1.3135350	-3.3127820
C	-5.8064120	0.1984780	-1.1986560
H	-6.3213930	0.1385800	-2.1676480
H	-6.1109990	-0.6758030	-0.6124400
H	-6.1860950	1.0921670	-0.6869910
C	-5.1399060	-0.1883690	2.3336970
H	-5.9624240	0.0235530	1.6426800
H	-5.5202680	-0.8818320	3.0963560
H	-4.8875220	0.7501610	2.8421850
C	-2.8142530	-1.1084490	2.6677540
H	-1.9561820	-1.6200680	2.2223410
H	-2.4426740	-0.1963440	3.1520160
H	-3.2174000	-1.7546990	3.4596960

Framework group C_1, energy: -1592.67806574 a.u.

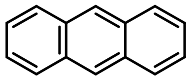 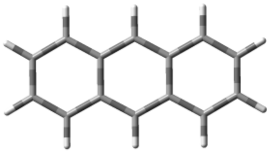

Atomic Type	X	Coordinates (Angstroms) Y	Z
C	0.0000000	3.7018680	0.6951530
C	0.0000000	2.4795660	1.3995530
C	0.0000000	1.2441460	0.7219810
C	0.0000000	1.2441460	-0.7219810
C	0.0000000	2.4795660	-1.3995530
C	0.0000000	3.7018680	-0.6951530
C	0.0000000	0.0000000	1.3971750
C	0.0000000	0.0000000	-1.3971750
C	0.0000000	-1.2441460	-0.7219810
C	0.0000000	-1.2441460	0.7219810
C	0.0000000	-2.4795660	1.3995530
H	0.0000000	-2.4828400	2.4867650
C	0.0000000	-3.7018680	0.6951530
C	0.0000000	-3.7018680	-0.6951530
C	0.0000000	-2.4795660	-1.3995530
H	0.0000000	0.0000000	2.4856690
H	0.0000000	4.6383740	1.2452450
H	0.0000000	2.4828400	2.4867650
H	0.0000000	2.4828400	-2.4867650
H	0.0000000	4.6383740	-1.2452450
H	0.0000000	0.0000000	-2.4856690
H	0.0000000	-4.6383740	1.2452450
H	0.0000000	-4.6383740	-1.2452450
H	0.0000000	-2.4828400	-2.4867650

Framework group D_{2h}, energy: -539.52275255 a.u.

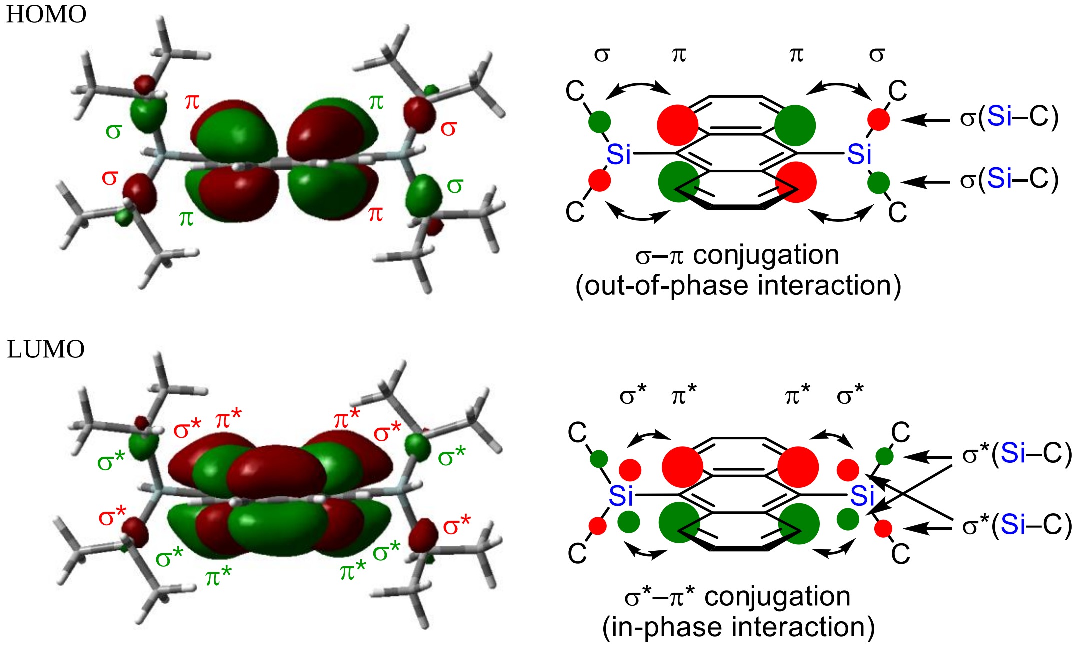

Figure S1. Side views of the HOMO (top) and LUMO (bottom) of the S_0 state of **1** calculated at the B3LYP/6-31G(d) level. The isovalue is 0.02. The lobes of the σ(Si–C) and σ*(Si–C) orbitals were explained in reference S3.

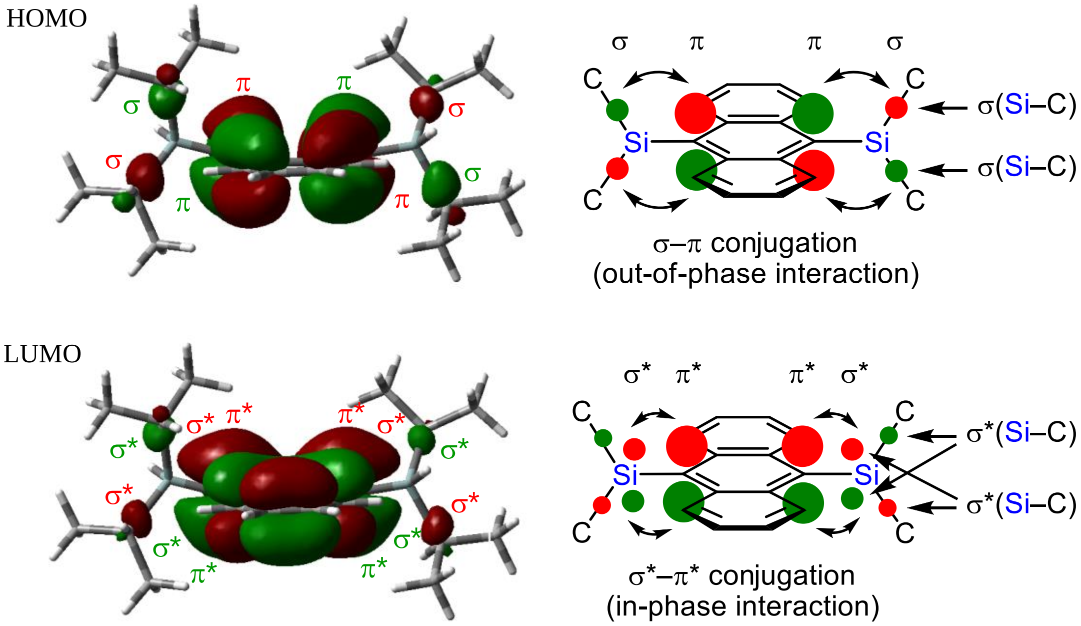

Figure S2. Side views of the HOMO (top) and LUMO (bottom) of the S_1 state of **1** calculated at the B3LYP/6-31G(d) level. The isovalue is 0.02.

Table S3. Transition energies, wavelengths and oscillator strengths of the transitions of the optimized structures of the S_0 states of **1** and anthracene.

Compound 1 [1]

Excited State 1: Singlet-A 3.0762 eV 403.04 nm f=0.1597 <S**2>=0.000
111 ->112 0.70135

Excited State 2: Singlet-A 3.8028 eV 326.03 nm f=0.0055 <S**2>=0.000
110 ->112 0.53191
111 ->113 0.46336

Excited State 3: Singlet-A 4.3671 eV 283.90 nm f=0.0000 <S**2>=0.000
109 ->112 0.66369
111 ->114 -0.23303

Excited State 4: Singlet-A 4.6416 eV 267.11 nm f=0.0057 <S**2>=0.000
104 ->112 -0.11614
106 ->112 0.66357
107 ->112 -0.20311

Excited State 5: Singlet-A 4.6857 eV 264.60 nm f=0.0191 <S**2>=0.000
108 ->112 0.65082
111 ->115 0.26685

Excited State 6: Singlet-A 4.7872 eV 258.99 nm f=0.0007 <S**2>=0.000
106 ->112 0.20267
107 ->112 0.66985

Excited State 7: Singlet-A 4.8512 eV 255.58 nm f=0.0030 <S**2>=0.000
109 ->112 0.21949
111 ->114 0.64705

Excited State 8: Singlet-A 4.8627 eV 254.97 nm f=0.0936 <S**2>=0.000
103 ->112 -0.13155
108 ->112 -0.24683
110 ->112 0.13244
111 ->113 -0.16944
111 ->114 -0.10557
111 ->115 0.59419

Excited State 9: Singlet-A 5.0731 eV 244.40 nm f=1.1422 <S**2>=0.000
110 ->112 -0.44077
111 ->113 0.50079

111 ->115 0.18553

Excited State 10: Singlet-A 5.2327 eV 236.94 nm f=0.0065 <S**2>=0.000
105 ->112 0.70063

Excited State 11: Singlet-A 5.3649 eV 231.10 nm f=0.0059 <S**2>=0.000
104 ->112 0.69294
106 ->112 0.11745

Excited State 12: Singlet-A 5.5264 eV 224.35 nm f=0.0000 <S**2>=0.000
102 ->112 -0.37017
109 ->113 0.46987
110 ->114 0.31687
111 ->116 -0.18111

Excited State 13: Singlet-A 5.7342 eV 216.22 nm f=0.0187 <S**2>=0.000
103 ->112 0.60740
109 ->114 0.11801
110 ->113 -0.28550
111 ->115 0.15437

Excited State 14: Singlet-A 5.8451 eV 212.12 nm f=0.1772 <S**2>=0.000
103 ->112 0.29530
109 ->114 -0.15022
110 ->113 0.59213

Excited State 15: Singlet-A 5.8610 eV 211.54 nm f=0.0048 <S**2>=0.000
102 ->112 0.50749
109 ->113 0.20844
110 ->114 0.38736
111 ->116 0.17236

Excited State 16: Singlet-A 6.0315 eV 205.56 nm f=0.0087 <S**2>=0.000
103 ->113 0.14151
108 ->113 -0.39332
110 ->115 0.54948

Excited State 17: Singlet-A 6.0445 eV 205.12 nm f=0.0011 <S**2>=0.000
101 ->112 0.67723

Excited State 18: Singlet-A 6.0808 eV 203.89 nm f=0.0008 <S**2>=0.000
97 ->112 0.12098
98 ->112 -0.21037

99 ->112 -0.13559
100 ->112 0.14269
104 ->113 -0.11106
106 ->113 0.59136
107 ->113 -0.14578

Excited State 19: Singlet-A 6.1332 eV 202.15 nm f=0.0010 <S**2>=0.000
96 ->112 0.11528
97 ->112 -0.21659
98 ->112 0.37361
99 ->112 0.24210
100 ->112 -0.25780
106 ->113 0.28243
107 ->113 -0.10275
109 ->113 -0.10428
111 ->116 -0.20355

Excited State 20: Singlet-A 6.1374 eV 202.01 nm f=0.0002 <S**2>=0.000
98 ->112 0.14266
102 ->112 -0.11227
106 ->113 0.13224
109 ->113 0.28092
110 ->114 -0.24328
111 ->116 0.52602

[1] The 111st orbital is the HOMO, and the 112nd orbital is the LUMO.

Anthracene [2]

Excited State 1: Singlet-B1U 3.2757 eV 378.50 nm f=0.0583 <S**2>=0.000
47 -> 48 0.69994

Excited State 2: Singlet-B2U 3.9082 eV 317.24 nm f=0.0017 <S**2>=0.000
46 -> 48 0.51330
47 -> 49 0.48418

Excited State 3: Singlet-B3G 4.5890 eV 270.18 nm f=0.0000 <S**2>=0.000
45 -> 48 0.58568
47 -> 50 -0.39218

Excited State 4: Singlet-B3G 4.9913 eV 248.40 nm f=0.0000 <S**2>=0.000
45 -> 48 0.38351
47 -> 50 0.57878

```
Excited State   5:      Singlet-B2U    5.3101 eV  233.49 nm  f=1.9082  <S**2>=0.000
      46 -> 48        -0.48671
      47 -> 49         0.51584

Excited State   6:      Singlet-B1U    5.5305 eV  224.18 nm  f=0.0000  <S**2>=0.000
      44 -> 48         0.50401
      47 -> 51        -0.49556

Excited State   7:      Singlet-AG     5.6006 eV  221.38 nm  f=0.0000  <S**2>=0.000
      43 -> 48         0.32331
      45 -> 49         0.48127
      46 -> 50         0.36557
      47 -> 52         0.16793

Excited State   8:      Singlet-B1U    5.8890 eV  210.54 nm  f=0.0797  <S**2>=0.000
      45 -> 50        -0.21243
      46 -> 49         0.65523

Excited State   9:      Singlet-AG     5.9804 eV  207.32 nm  f=0.0000  <S**2>=0.000
      43 -> 48         0.54220
      45 -> 49        -0.10877
      46 -> 50        -0.40813
      47 -> 52         0.16094

Excited State  10:      Singlet-B3U    6.1351 eV  202.09 nm  f=0.0000  <S**2>=0.000
      42 -> 48         0.70256

Excited State  11:      Singlet-B1U    6.2322 eV  198.94 nm  f=0.0215  <S**2>=0.000
      44 -> 48         0.47371
      46 -> 49         0.15065
      47 -> 51         0.48563

Excited State  12:      Singlet-AG     6.3039 eV  196.68 nm  f=0.0000  <S**2>=0.000
      45 -> 49        -0.40284
      46 -> 50         0.30280
      47 -> 52         0.49347

Excited State  13:      Singlet-AU     6.3596 eV  194.96 nm  f=0.0000  <S**2>=0.000
      41 -> 48         0.70214

Excited State  14:      Singlet-B2U    6.6911 eV  185.30 nm  f=0.0003  <S**2>=0.000
      43 -> 50        -0.10240
      44 -> 49         0.47474
```

46 -> 51 0.50266

Excited State 15: Singlet-B1G 6.7419 eV 183.90 nm f=0.0000 <S**2>=0.000
40 -> 48 0.69515
42 -> 50 -0.11370

Excited State 16: Singlet-B1U 6.9606 eV 178.12 nm f=0.2741 <S**2>=0.000
44 -> 48 -0.10407
45 -> 50 0.66137
46 -> 49 0.17551

Excited State 17: Singlet-B2G 7.1017 eV 174.58 nm f=0.0000 <S**2>=0.000
47 -> 53 0.69765

Excited State 18: Singlet-AU 7.2093 eV 171.98 nm f=0.0000 <S**2>=0.000
45 -> 53 -0.10322
47 -> 54 0.69749

Excited State 19: Singlet-B2U 7.2343 eV 171.38 nm f=0.0012 <S**2>=0.000
39 -> 48 0.46840
44 -> 49 0.34131
46 -> 51 -0.39207

Excited State 20: Singlet-B3G 7.2797 eV 170.32 nm f=0.0000 <S**2>=0.000
43 -> 49 0.64138
44 -> 50 0.11798
45 -> 51 0.19474
46 -> 52 -0.18558

[2] The 47th orbital is the HOMO, and the 48th orbital is the LUMO.

Table S4. Transition energies, wavelengths and oscillator strengths of the transitions of the optimized structures of the S_1 states of **1** and anthracene.

Compound 1 [1]

Excited State	1:	Singlet-A	2.6333 eV	470.84 nm	f=0.1559	<S**2>=0.000
111 ->112		0.70569				
Excited State	2:	Singlet-A	3.6546 eV	339.25 nm	f=0.0099	<S**2>=0.000
110 ->112		0.54684				
111 ->113		0.44544				
Excited State	3:	Singlet-A	3.9745 eV	311.95 nm	f=0.0000	<S**2>=0.000
109 ->112		0.67190				
111 ->114		0.20758				
Excited State	4:	Singlet-A	4.3041 eV	288.06 nm	f=0.0159	<S**2>=0.000
106 ->112		0.30394				
107 ->112		0.19221				
108 ->112		0.58313				
111 ->115		0.14617				
Excited State	5:	Singlet-A	4.3455 eV	285.31 nm	f=0.0182	<S**2>=0.000
106 ->112		-0.39338				
107 ->112		-0.45036				
108 ->112		0.33462				
111 ->115		0.12535				
Excited State	6:	Singlet-A	4.4559 eV	278.24 nm	f=0.0046	<S**2>=0.000
106 ->112		-0.47896				
107 ->112		0.50040				
Excited State	7:	Singlet-A	4.5651 eV	271.59 nm	f=0.0002	<S**2>=0.000
109 ->112		0.19745				
111 ->114		-0.66529				
Excited State	8:	Singlet-A	4.6094 eV	268.98 nm	f=0.0488	<S**2>=0.000
103 ->112		-0.14065				
105 ->112		-0.20588				
108 ->112		-0.17703				
111 ->113		-0.12724				
111 ->115		0.61248				
Excited State	9:	Singlet-A	4.8611 eV	255.06 nm	f=0.2628	<S**2>=0.000

103 ->112 -0.14021
105 ->112 0.55474
110 ->112 -0.21891
111 ->113 0.25910
111 ->115 0.22243

Excited State 10: Singlet-A 4.9050 eV 252.77 nm f=0.8312 <S**2>=0.000
105 ->112 0.37149
110 ->112 0.37369
111 ->113 -0.46001

Excited State 11: Singlet-A 5.0554 eV 245.25 nm f=0.0062 <S**2>=0.000
104 ->112 0.68691
106 ->112 -0.12361

Excited State 12: Singlet-A 5.3348 eV 232.41 nm f=0.0011 <S**2>=0.000
102 ->112 -0.52598
109 ->113 0.28346
110 ->114 -0.15353
111 ->116 0.31555

Excited State 13: Singlet-A 5.4069 eV 229.31 nm f=0.0572 <S**2>=0.000
103 ->112 0.65954
111 ->115 0.17861

Excited State 14: Singlet-A 5.7194 eV 216.78 nm f=0.0012 <S**2>=0.000
101 ->112 0.57860
102 ->112 0.18747
109 ->113 0.20989
110 ->114 -0.24248

Excited State 15: Singlet-A 5.7503 eV 215.61 nm f=0.0006 <S**2>=0.000
101 ->112 0.34657
102 ->112 -0.20737
109 ->113 -0.39346
110 ->114 0.36641
111 ->116 0.18821

Excited State 16: Singlet-A 5.8242 eV 212.88 nm f=0.0032 <S**2>=0.000
96 ->112 0.66483
99 ->112 -0.11418
100 ->112 -0.11345

Excited State 17: Singlet-A 5.8435 eV 212.18 nm f=0.0054 <S**2>=0.000
101 ->112 0.12191
102 ->112 -0.27532
109 ->113 0.18579
110 ->114 0.20183
111 ->116 -0.55295

Excited State 18: Singlet-A 5.9715 eV 207.63 nm f=0.0815 <S**2>=0.000
109 ->114 0.25459
110 ->113 0.62214
110 ->115 -0.13651

Excited State 19: Singlet-A 6.0701 eV 204.25 nm f=0.0062 <S**2>=0.000
103 ->113 0.11584
108 ->113 -0.43425
110 ->113 0.14698
110 ->115 0.49294

Excited State 20: Singlet-A 6.1384 eV 201.98 nm f=0.0032 <S**2>=0.000
95 ->112 -0.10996
109 ->113 -0.14792
110 ->114 -0.15055
111 ->117 0.64670

[1] The 111st orbital is the HOMO, and the 112nd orbital is the LUMO.

Anthracene [2]

Excited State 1: Singlet-B1U 2.8491 eV 435.17 nm f=0.0601 <S**2>=0.000
47 -> 48 0.70468

Excited State 2: Singlet-B2U 3.7782 eV 328.16 nm f=0.0009 <S**2>=0.000
46 -> 48 0.51051
47 -> 49 -0.48705

Excited State 3: Singlet-B3G 4.2294 eV 293.15 nm f=0.0000 <S**2>=0.000
45 -> 48 0.58547
47 -> 50 -0.39309

Excited State 4: Singlet-B3G 4.6982 eV 263.90 nm f=0.0000 <S**2>=0.000
45 -> 48 0.38514
47 -> 50 0.57877

Excited State 5: Singlet-B2U 5.1952 eV 238.65 nm f=1.9229 <S**2>=0.000

46 -> 48 0.49071
47 -> 49 0.51402

Excited State 6: Singlet-B1U 5.2358 eV 236.80 nm f=0.0000 <S**2>=0.000
44 -> 48 0.53376
47 -> 51 -0.46332

Excited State 7: Singlet-AG 5.4582 eV 227.15 nm f=0.0000 <S**2>=0.000
43 -> 48 0.49780
45 -> 49 -0.36758
46 -> 50 0.21930
47 -> 52 0.25976

Excited State 8: Singlet-AG 5.8438 eV 212.17 nm f=0.0000 <S**2>=0.000
43 -> 48 -0.38704
45 -> 49 -0.33489
46 -> 50 0.46769
47 -> 52 -0.13052

Excited State 9: Singlet-B3U 5.8873 eV 210.60 nm f=0.0001 <S**2>=0.000
42 -> 48 0.70336

Excited State 10: Singlet-B1U 5.9252 eV 209.25 nm f=0.0016 <S**2>=0.000
44 -> 48 0.37740
45 -> 50 0.25179
46 -> 49 0.31003
47 -> 51 0.44194

Excited State 11: Singlet-AU 6.0898 eV 203.59 nm f=0.0000 <S**2>=0.000
41 -> 48 0.70242

Excited State 12: Singlet-B1U 6.0965 eV 203.37 nm f=0.0570 <S**2>=0.000
44 -> 48 -0.24640
45 -> 50 0.16689
46 -> 49 0.56710
47 -> 51 -0.28096

Excited State 13: Singlet-AG 6.1377 eV 202.01 nm f=0.0000 <S**2>=0.000
43 -> 48 -0.12925
45 -> 49 0.36276
46 -> 50 0.29530
47 -> 52 0.51194

Excited State 14: Singlet-B1G 6.5678 eV 188.78 nm f=0.0000 <S**2>=0.000
40 -> 48 0.69684
42 -> 50 -0.10299

Excited State 15: Singlet-B2U 6.7491 eV 183.70 nm f=0.0005 <S**2>=0.000
39 -> 48 -0.12435
43 -> 50 0.13705
44 -> 49 0.50921
45 -> 52 0.10993
46 -> 51 -0.43848

Excited State 16: Singlet-B1U 6.8042 eV 182.22 nm f=0.3062 <S**2>=0.000
45 -> 50 0.63242
46 -> 49 -0.25902

Excited State 17: Singlet-B2G 6.9127 eV 179.36 nm f=0.0000 <S**2>=0.000
47 -> 53 0.69838

Excited State 18: Singlet-AU 6.9980 eV 177.17 nm f=0.0000 <S**2>=0.000
47 -> 54 0.69938

Excited State 19: Singlet-B2U 7.1032 eV 174.55 nm f=0.0055 <S**2>=0.000
39 -> 48 0.62073
44 -> 49 -0.10559
46 -> 51 -0.30236

Excited State 20: Singlet-B3U 7.2035 eV 172.12 nm f=0.0000 <S**2>=0.000
47 -> 55 0.70388

[2] The 47th orbital is the HOMO, and the 48th orbital is the LUMO.

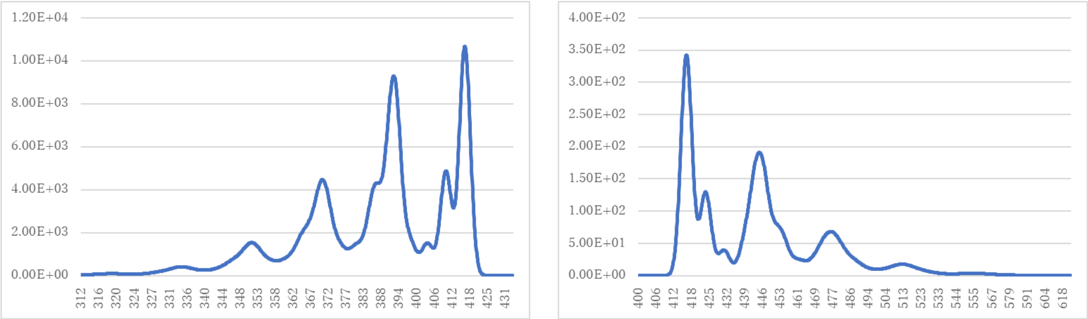

Figure S3. Vibrationally resolved UV/Vis (left) and fluorescence (right) spectra of anthracene calculated using the frequency data of the optimized structures of the S_0 and S_1 states.
